# Prevalence of Cutaneous Leishmaniasis in Districts of High and Low Endemicity in Mali

**DOI:** 10.1371/journal.pntd.0005141

**Published:** 2016-11-29

**Authors:** Bourama Traoré, Fabiano Oliveira, Ousmane Faye, Adama Dicko, Cheick A. Coulibaly, Ibrahim M. Sissoko, Samake Sibiry, Nafomon Sogoba, Moussa Brema Sangare, Yaya I. Coulibaly, Pierre Traore, Sekou F. Traore, Jennifer M. Anderson, Somita Keita, Jesus G. Valenzuela, Shaden Kamhawi, Seydou Doumbia

**Affiliations:** 1 International Center of excellence in research (ICER-MALI), University of Sciences, Techniques and Technology of Bamako (USTTB), Bamako, Mali; 2 Laboratory of Malaria and Vector Research (LMVR), National Institute of Allergy and Infectious Diseases (NIAID), National Institutes of Health (NIH), Rockville, Maryland, United States of America; 3 Centre National d’Appui à la Lutte contre la Maladie (CNAM), Bamako, Mali; Institute of Tropical Medicine, BELGIUM

## Abstract

Historically the western sahelian dry regions of Mali are known to be highly endemic for cutaneous leishmaniasis (CL) caused by *Leishmania major*, while cases are rarely reported from the Southern savanna forest of the country. Here, we report baseline prevalence of CL infection in 3 ecologically distinct districts of Mali (dry sahelian, north savanna and southern savanna forest areas). We screened 195 to 250 subjects from 50 to 60 randomly selected households in each of the 6 villages (four from the western sahelian district of Diema in Kayes region, one from the central district of Kolokani and one from the southern savanna district of Kolodieba, region of Sikasso). The screening consisted of: 1] A Leishmanin Skin Test (LST) for detection of exposure to *Leishmania* parasites; 2] clinical examination of suspected lesions, followed by validation with PCR and 3] finger prick blood sample to determine antibody levels to sand fly saliva. LST positivity was higher in the western district of Diema (49.9%) than in Kolokani (24.9%) and was much lower in Kolondieba (2.6%). LST positivity increased with age rising from 13.8% to 88% in Diema for age groups 2–5 years and 41–65 years, respectively. All eight PCR-confirmed *L*. *major* CL cases were diagnosed in subjects below 18 years of age and all were residents of the district of Diema. Exposure to sand fly bites, measured by anti-saliva antibody titers, was comparable in individuals living in all three districts. However, antibody titers were significantly higher in LST positive individuals (P<0.0001). In conclusion, CL transmission remains active in the western region of Mali where lesions were mainly prevalent among children under 18 years old. LST positivity correlated to higher levels of antibodies to sand fly salivary proteins, suggesting their potential as a risk marker for CL acquisition in Mali.

## Author Summary

It is generally assumed that neglected tropical diseases (NTDS) such as leishmaniasis are concentrated in poor populations. It affects as many as 12 million people, with 1.5 to 2 million new cases every year around the world. Depending on the species of *Leishmania*, the host can develop cutaneous leishmaniasis (CL) or visceral leishmaniasis. In Mali, CL caused by *Leishmania major* is transmitted through the bite of infected sand flies belonging to the species *Phlebotomus duboscqi*. The objectives of this study were to determine the baseline prevalence of LST positivity, a test of previous exposure to *Leishmania* parasites, the prevalence of CL lesions, and the level of anti-*P*. *duboscqi* salivary antibodies, indicative of exposure to vector bites, in populations living in western, central and southern Mali, three ecologically distinct study sites. LST positivity was higher in the western district of Diema (85.1%) than in Kolokani (24.6%) and was much lower in Kolondieba (2.7%). All eight PCR-confirmed *L*. *major* CL cases were diagnosed in subjects below 18 years of age and all were residents of the district of Diema. Exposure to sand fly bites, measured by anti-saliva antibody titers, was established in individuals living in these three districts and antibody titers were higher in LST positive individuals (P<0.0001). The finding of this study provides an update on the prevalence of CL in these regions.

## Introduction

Leishmaniasis is a disease caused by *Leishmania*, a protozoan transmitted to humans by the bite of the sand fly [1]. There are different forms and clinical manifestations of the disease that depend primarily on the *Leishmania* species incriminated. The major clinical manifestations of leishmaniasis are visceral, muco-cutaneous or cutaneous. CL is currently endemic in 87 countries worldwide [2] including 20 countries of the New World (South and Central America) and in 67 countries in the Old World (Europe, Africa, Middle East, central Asia and the Indian subcontinent). An estimated 500,000–1,000,000 new cases occur annually but only a small fraction of cases (19%–37%) are actually reported to health authorities [3]. In the Old World, cutaneous leishmaniasis (CL) is the most common form of the disease. Cutaneous leishmaniasis caused by *L*. *major* frequently appears as severely inflamed and ulcerated skin, which usually heals spontaneously within 2–8 months. It usually produces ulcers on the exposed parts of the body, such as the face, arms and legs. There may be multiple lesions, especially in non-immune patients, which can cause serious disability. When the ulcers heal, they invariably leave permanent scars, which are often the cause of serious social prejudice.[4] [5].

In Mali, *L*. *major* was identified as the predominant causative *Leishmania* species responsible for CL disease [6]. Moreover, *Phebotomus duboscqi* was incriminated as the main vector of *L*. *major* [7]. Although the reservoirs of CL in Mali have not been established, rodent species reported from the country are well known reservoirs for *L*. *major* throughout its distribution range in West Africa [8, 9]. Compared to other parts of the country, the region of Kayes in the western part of Mali is known for its higher endemicity for *Leishmania* infection [10, 11]. The last study using a leishmanin skin test (LST) to determine the prevalence of CL in Kayes region dates back to late 1960’s [12]. No data is available on the epidemiology of the disease in southern regions, while previous studies carried out in the central district of Baroueli, reported a LST positivity ranging from 20% to 45% [10]. The objectives of this study were to determine the baseline prevalence of LST positivity, the prevalence of CL lesions, and the level of anti-*P*. *duboscqi* salivary antibodies in populations living in western, central and southern Mali, three ecologically distinct study sites. The finding of this study provides an update on the prevalence of CL in these regions.

## Material & Methods

### Ethics statement

The study protocol (Protocol # 12–0075) was approved by the Institutional Review Boards (IRB) of the Faculty of Medicine, Pharmacy and Odontostomatology (FMPOS), Bamako, Mali, and of the National Institutes of Allergy and Infectious Diseases (USA).

A collective village-wide oral consent was obtained from the villages’ elders, and all adult participants signed individual written informed consent and a parent or guardian of any child participant provided informed consent on their behalf.

### Study setting

**Fig 1:**
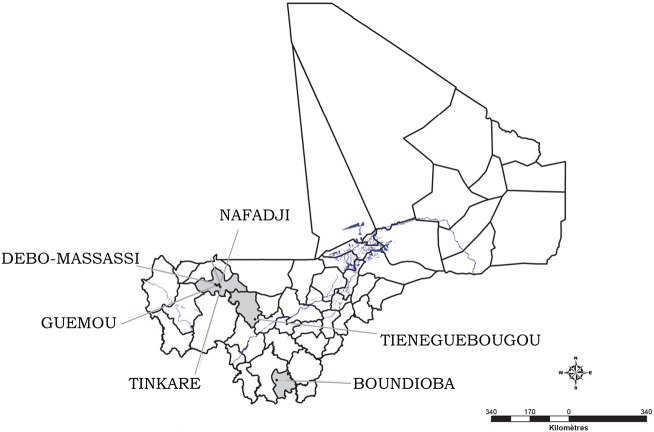
Map of Mali showing the study sites in different districts.

The study was carried out in June 2014 in three ecologically distinct districts (Fig 1): 1] The district of Diema in the Region of Kayes, located at 345 km from Bamako in the western part of the country. The study was carried out in 4 villages: Nafadji (9.233399W, 14.557710N), Guemou (9.312490W, 14.546290N), Debo Massassi (9.368320W, 14.579850N) and Tinkare (9.179729W, 14.490520N). In Diema, the undulating topography is dominated by sandy plains and a plateau with a few rocky outcrops, and is an extension of Mont Manding. The climate is typical Sahelian with sandy clayey soil, characterized by the alternation of two seasons with temperatures varying between 15°C and 45°C depending on the season. The rainy season is short lasting from July to October, with rainfall ranging from 400 mm to 800 mm. The dry season lasts from November to June. The site is influenced by the harmattan, a dry wind that blows from the northeast to the southwest, and the monsoon bringing rain. The vegetation is characterized by shrub and tree vegetation. The population is composed mainly of Sarakolé, Bambara, Peulh, Moor, and Kagoro ethnicities.

2] The district of Kolokani, in the region of Koulikoro, is located at 105 km northwest of Bamako in central Mali. The study was carried out in the village of Tieneguebougou (8.077450W, 13.57639N). The north of the district is dry, Sahel land, primarily used for livestock. The study site is at the interface between the sahelian and the wetter Sudan to the south. The population is composed mainly of Bambara, Peulh, Mossi, and Dioula ethnicities

3] The district of Kolondieba, region of Sikasso, located at 250 km from Bamako in the southern part of Mali. The study was carried out in the village of Boundioba (6.982890W, 11.040190N). The climate is typically south-savannah type with clear forests and an average of 1250 mm of rain spread over 60 days per year. The average temperatures vary from 20°C to 31°C in the same year. The population is composed of Bambara, Peulh, Senoufos and Sarakolés ethnicities.

Based on historical data from the National referral dermatologic hospital of Bamako, CL cases are regularly recorded from Diema, but no population-based prevalence of CL is available. The districts of Kolokani and Kolondieba are known to be endemic for lymphatic filarial (LF) but not for CL.

### Study population and sampling

In each village, 40 to 50 households were randomly selected (with an average of 5–7 persons per household, and 195 to 250 subjects per village) and screened for CL. Households were randomly selected from a list obtained from a census data collected by the study team. Subjects living in the randomly selected households were included in the study: 2–65 years old in the district of Diema (Nafadji, Guemou, Debo Massassi and Tinkare), and 18–65 years old in the districts of Kolokani (Tieneguebougou) and Kolondieba (Boundioba). We targeted adults for recruitment in the districts of Kolokani and Kolondieba due to their low endemicity for CL. After informed consent, all members of the selected households were invited to participate the screening. The screening consisted of clinical examination by a dermatologist, a LST and a finger prick to collect blood samples for immunological studies.

#### Leishmanin skin test (LST)

The LST (leishmanin, IRC 1228181375, LOT #127; Institute Pasteur of Iran, Tehran) was performed at the beginning of the study as described elsewhere [10]. Briefly, 0.1 ml of leishmanin was injected intradermally in the left forearm. Each ml of preparation contains, 6×10^6^ killed *L*. *major* promastigotes (MRHO/IR/75/ER strain) and Thimerosal 0.01% in phosphate buffered saline (PBS) at pH 7.0. Readings were taken 48 to 72 hours after the injection using the ballpoint-pen technique. The induration was measured in two perpendicular directions. A mean of the two measurements of 5 mm or greater was considered as positive [13]. In the case of no reactivity, the test would be observed at 72 h of the injection. All subjects who were not seen by 72 hours post injection of leishmanin were excluded.

#### Blood samples collection

Capillary blood samples were collected for anti-sand fly salivary proteins antibody measurement from all study participants screened by LST in each village. The blood collection was made right after LST testing. The finger of each participant was cleaned with isopropyl alcohol and pricked with a sterile single use disposable lancet. A maximum of 5 drops of whole blood (~ 50 μL per drop, 250 μL total) were dropped onto filter papers (Whatman 903 Protein Saver Cards)[14] labeled with the participant’s unique identification number. Thereafter, a subset of fifty individuals out of the 250 collected samples were randomly chosen to test for levels of anti-sand fly saliva antibodies tested.

#### Anti-saliva antibodies by ELISA

Filter paper containing the blood sample was obtained using a 6mm disposable punch and eluted in PBS 0.05% Tween and kept at room temperature (RT) overnight. ELISA was performed as described elsewhere [15]. Briefly, ELISA plates were coated with 50 μl of salivary gland homogenate of *P*. *duboscqi* diluted to 2μg/ml in Carbonate/Bicarbonate buffer (NaHCO3 0.45 M, Na2CO3 0.02 M, pH 9.6) overnight at 4°C. After three washes with PBS- 0.05% Tween, the plates were blocked with PBS containing 4% bovine serum albumin for 2 hours at room temperature. The plates were washed three times with PBS 0.05% Tween, the sera were added and incubated at RT for one hour. After further washing, the antibody Goat anti-human IgG (H+L) alkaline phosphatase conjugated (Sigma, MO) were added and incubated at 37° for one hour. Following another washing, *p-nitrophenyl phosphate* substrate (Sigma Sr. Louis, MO) was added and the absorbance was read at 405nm on a Versamax microplate reader after 30 minutes. Values obtained were subtracted from those obtained for the background (i.e. OD values for wells where PBS was added instead of eluted blood).

#### Active detection of CL in individuals

Interviews and clinical examination were conducted through house-to-house visits by experienced dermatologists to screen for any suspected skin lesions. Interviews included the history of the lesion, while physical examination provided information on the location (head/neck, trunk, upper extremity, lower extremity) and duration (in days up to the date of the visit) of each lesion. Lesions were documented through photography. Non-invasive scrapings of the border of the lesions were performed and tested by both PCR and microscopy to confirm the presence of *L*. *major*. All subjects diagnosed with CL, by PCR or microscopy, were treated with Meglumine Antimoniate.

#### Detection of *Leishmania major*

Parasite DNA from suspected lesions was extracted using QIAamp DNA Micro Kit according to the manufacturer’s instructions [16], and stored at -20°C until PCR amplification. Leishmania DNA was detected by PCR using forward and reverse primers for *Leishmania sp*. (Uni21/Lmj4) as described in [17]. The primer design was based on the published Kinetoplastid DNA minicercle sequence (kDNA) from *L*. *major* [18].

### Statistical analysis

The data were recorded on a case form (CRF), entered in iDataFax management (Version 2014.1.0), and analyzed using the Statistical Package for the Social Sciences (SPSS, Chicago, IL, USA). Descriptive analyses were used to assess the association between LST and demographic variables. Fisher's exact test was used to assess the association between infection and demographic variables. Age means between villages were compared using an independent samples t-test. One-way ANOVA with Bonferroni's multiple comparison test was applied to evaluate the difference in the mean size of the LST reaction between the two study sites.

## Results

Overall, 1,428 volunteers of both sexes and different age groups were enrolled. The overall median age of the participants was 23 years, and 62.1% were female. The relative frequencies of the different age groups within the study population per study site are provided in Table 1. The highest mean age of study subjects was observed in Tiénekebougou (37.55±13.34 years) and the lowest in Tinkare (14.58±14.07 years).

**Table 1:** Age distribution of enrolled participants, by districts and by sites.

		Age groups				
Districts	Sites	[2–5] (%)	[6–18] (%)	[19–40] (%)	[41–65] (%)	Total
**Diéma**	Nafadji	29 (11.6)	120 (48.0)	77 (30.8)	24 (09.6)	250
	Guemou	44 (17.7)	111 (44.8)	77 (31.0)	16 (06.5)	248
	Debo Massassi	57 (22.8)	96 (38.4)	77 (30.8)	20 (08.0)	250
	Tinkare	62 (24.8)	131 (52.4)	38 (15.2)	19 (07.6)	250
**Kolokani**	Tiénekebougou	NA	NA	107 (57.5)	79 (42.5)	186
**Kolondieba**	Boundioba	NA	NA	139 (69.9)	89 (39.0)	228
	**Total**			515 (36.1)	247 (17.3)	1412

### Prevalence of Leishmanin skin test positivity

**Fig 2:**
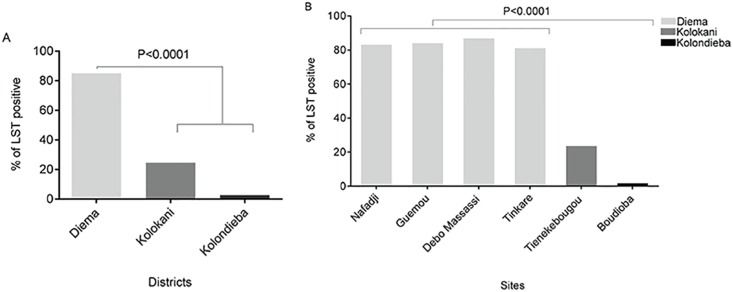
Prevalence of LST positivity by district (A) and by site (B).

Significant differences in LST positivity (LST+) per district were observed among individuals in the age groups 19–40 and 41–65 years of age (Fig 2A). The highest LST+ was observed in Diema (85.1%) followed by Kolokani (24.6%) and was lowest at 2.7% in Kolondieba. Compared to Kolokani and Kolondieba, a significantly higher prevalence of LST positivity was observed for each of the study villages of the district of Diema (Fig 2B). Moreover, the high prevalence rates of LST+ were comparable among 3 of the 4 villages (Nafadji, Guemou, Debo Massassi) of the district of Diema (Fig 2B).

**Fig 3:**
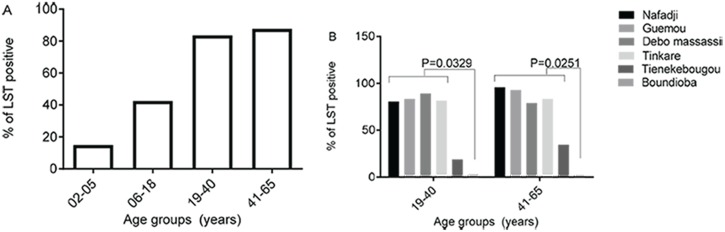
Prevalence of LST positivity by age group for the district of Diema (A) and for all study sites (B).

In the district of Diema, the percentage of participants with a positive LST increased steadily with age at 13.8% [9.2–19.5], 41.3% [36.3–46.0] and 83.9% [78.4–94.4] for age groups 2–5 years, 6–18 years, 19–40 years and 41–65 years, respectively (Fig 3A, Table 2). The high LST positivity in the district of Diema was stable among its four tested villages, and was greater than the positivity observed in Tienekebougou and Boundioba villages (Fig 3B). Moreover, it was significantly different among age groups 19–40 years (P = 0.0329) and 41–65 years (P = 0.0251) between the site of Boundioba compared to the four sites of Nafadji, Guemou, Debo massassi and Tinkare (Fig 3B). In the district of Kolondieba, the prevalence of LST positivity was low and stable (around 2–3%) across age groups (Fig 3B).

**Table 2:** Prevalence of LST positivity by age group and by study districts.

Group age	Diema		Kolokani		Kolondieba		Total	
	n(N)	% [95% CI]	n(N)	% [95% CI]	n(N)	% [95% CI]	n(N)	% [95% CI]
**[2**–**5]**	26(189)	**13.8 [9.2–19.5]**	NA	NA	NA	NA	26(190)	**13.7 [NA]**
**[6**–**18]**	188(455)	**41.3 [36.8–46.0]**	NA	NA	NA	NA	189(470)	**40.2 [NA]**
**[19–40]**	214(255)	**83.9 [88.2–78.8]**	20(106)	**18,9 [11.9–27.6]**	4(139)	**2.9 [NA]**	238(500)	**47.6 [NA]**
**[41–65]**	66(75)	**88 [78.4–94.4]**	27(78)	**34.6 [24.2–46.2]**	2(89)	**2.2 [NA]**	95(242)	**39.3 [NA]**
**Total**	494(974)	**50.7 [47.5–53.9]**	47(185)	**25.4 [18.9–31.6]**	6(228)	**2.6 [NA]**	548(1402*)	**39.1 [NA]**

* 10 subjects were lost to follow-up

n = the number of LST positive participants; N = the total number of participants

CI = Confidence Interval; NA = Not Application

### Assessing Anti-saliva antibodies levels

**Fig 4:**
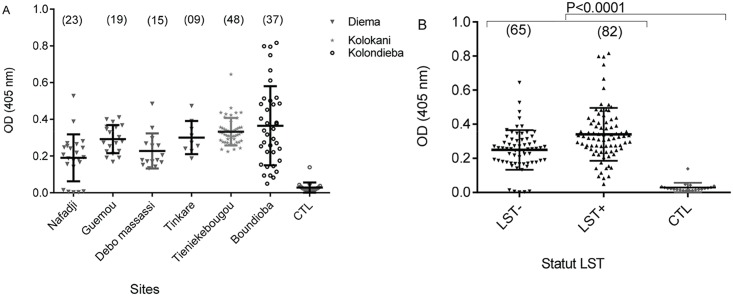
Optical density (OD) of antibodies against sand fly saliva measured by ELISA. A) Antibody levels in tested subjects from the 6 study villages. Sample size is shown in brackets. B) Overall antibody levels in leishmanin positive (LST+) and negative (LST-) study subjects. CTL, non-endemic controls from US healthy volunteers not exposed to *Leishmania*.

Levels of *P*. *duboscqi* saliva specific IgG antibodies were similar in all the study sites and were significantly higher (P<0.0001) compared to non-endemic controls (US healthy volunteers with no previous history of exposure to *Leishmania*) (Fig 4A). Moreover, the median value of anti-salivary IgG levels were not significant in LST+ compared to LST- study subjects (Fig 4B).

### Prevalence of CL lesion in the study areas

**Fig 5:**
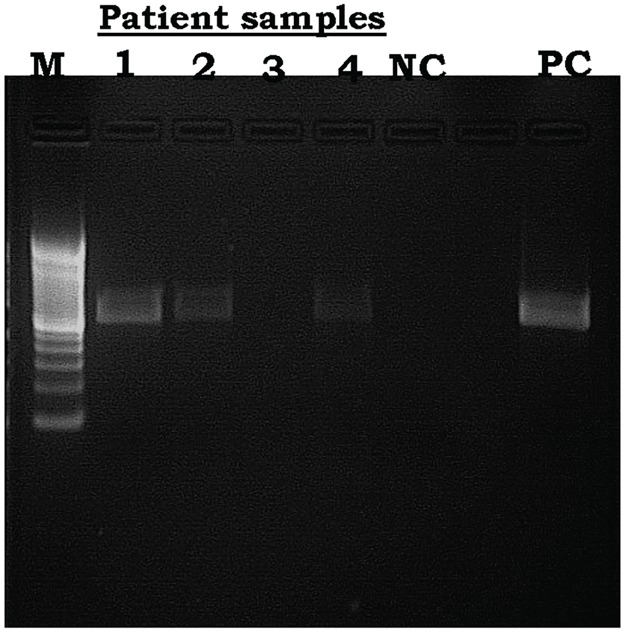
*Leishmania major* detection from four of the 11 tissue aspirates obtained from lesions of active CL cases. M = ladder 100bp, lanes 1–4 = samples, NC = negative control, PC = Positive Control (*L*. *major*).

During active case detection, 11 suspected lesions were found, of which 8 were confirmed by PCR amplification (Fig 5) and microscopy. The positive cases were from district of Diema, six cases in Nafadji, one in Guemou and one in Tinkare.

**Fig 6:**
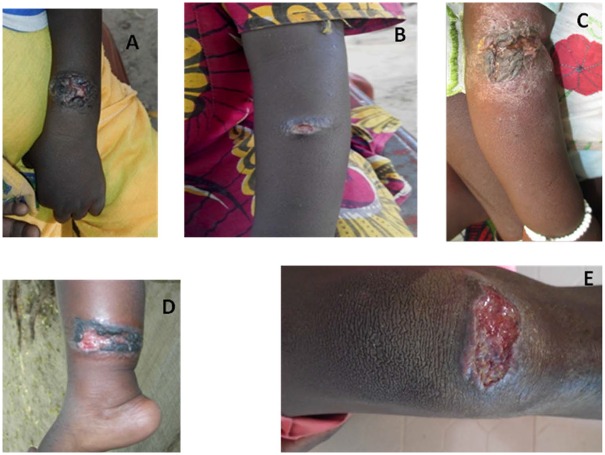
Images representative of active CL cases diagnosed during the study. **A**: Ulcero-crusted lesion of CL (3 to 4 cm diameter) on the forearm of 8 years old girl from the village of Nafadji. **B**: Ulcerated nodule of CL (1.5 cm diameter) of the left forearm of 10 years old girl from the village of Nafadji. **C**: Superinfected and crusted lesion of CL of the right forearm of a 3 years old girl from the village of Guemou. Please note the dry and erythematous halo with an eczematization of the lesion. **D**: Ulcerated lesion of CL with black crusted lesion (covered by a local traditional powder) of leg of a 2 years boy from the village of Nafadji. **E**: Ulcerated nodule of CL of the right knee of a 13 years old boy from the village of Tinkare.

The lesions were clinically characterized as simple or multiple ulcero-crusted lesions, and were mostly observed on the limbs and forehead (Fig 6, Table 3). All eight patients positive for CL were treated with Meglumine Antimoniate (GLUCANTIME) without complications. The mean age of confirmed CL cases were 6.5±5.35 years. The minimum age was 2 years and the maximum was 17 years (Table 3).

**Table 3:** Characteristics of active CL cases diagnosed during the study.

Age groups	Sex		No of cases	No. of lesions		Mean duration* (Days)	PCR+
	Female	Male		Unique	Multiple		
≤5	1	4	5	4	1	120	5
6–10	3	0	3	2	1	120	2
≥11	0	3	3	2	1	165	1
Total	4	7	11	8	3	-	8

*up to diagnosis

## Discussion

LST has been used for years as an aid for diagnosis, and in epidemiological studies for assessing exposure to Leishmania infection [5, 9, 10]. LST remains an important tool in measurement of delayed-type hypersensitivity reactions (DTH) and consequently in assessment of cell-mediated immunity. It plays a major role in defining the immunity status of volunteers to leishmaniasis in vaccine trials [19], the assessment of vaccine efficacy, and the effectiveness of vaccination [20] [21]. In this cross-sectional study we present results of LST positivity in three different climatic areas of Mali in the districts of Diema, Kolokani and Kolondieba in western, central and southeast Mali, respectively. Our data showed a higher prevalence of LST+ in all the villages located in the Sahelian district of Diema compared to those in the district of Kolokani in central Mali and Kolondieba in southern Mali. Indeed, the overall prevalence of LST in ages 19 to 65 years was 84.2% (N = 101) in Nafadji, 85.0% (N = 80) in Guemou, 87.9% (N = 91) in Debo Massassi and 82.1% (N = 56) in Tinkare. In Tiénekebougou located in the district of Kolokani, central Mali, LST prevalence was 24.6% (N = 175), while in Boundioba in the district of Kolondieba, southern Mali, it was only 2.7% (N = 224). This north to south decrease in the prevalence of LST+ can be partly explained by the same pattern of decrease observed in the densities of *P*. *duboscqi*, the *Phlebotomus* species incriminated in the transmission of CL in Mali [13]. In a study carried out ten years ago in Baroueli, a neighboring district of Kolokani, Oliveira et al [10] observed a LST positivity ranging from 45.4% to 19.9% suggesting that transmission is stable in central Mali.

Our data also showed that the prevalence of LST positivity increased with age in Diema and Kolokani districts, consistent with other studies [10, 22, 23]. This age pattern was not observed in Boundioba probably because of the low exposure of the population to infected sand fly bites compared to Diema, and at a lesser extent to Kolokani. Interestingly, the overall mean anti-saliva specific IgG antibody level was similar in all study districts but was significantly higher (P<0.0001) in LST positive compared to LST negative subjects. This result is in accordance with previous studies using salivary proteins from *Lutzomyia longipalpis* [15] and *P*. *sergenti* [24] where anti-saliva antibody levels positively correlated with previous exposure to *Leishmania* parasites.

Similar to previous reports from Mali [6, 7, 25, 26], all active cases of CL identified during this study were caused by *L*. *major*. Though *L*. *major* is a self-healing disease, the diagnosed lesions were mostly large, disfiguring and ulcerative in nature reflecting the need for surveillance, early treatment and control. All the confirmed cases were from the western part of Mali, in the district of Diema corroborating data from the current LST survey as well as previous studies [6, 11, 12, 27] of its high endemicity for CL. Six of the eight confirmed CL cases in this locality were from Nafadji, and the rest were from Guemou and Tinkare. The active cases were mostly in children, the oldest patient being 17 years of age. This suggests that transmission is peridomestic where children are exposed to bites of infected sand flies. Additionally, children may be more exposed to sand fly bites due to the way they dress as well as the nature of their activities such as shepherding animals in pastures. Similar to previous reports from other parts of the country [10, 28], no active CL cases were detected from the districts of Kolokani and the district of Kolondieba despite a evidence of exposure to *Leishmania* by LST of 24.6% and 2.7%, respectively. The presence of *P*. *duboscqi* anti-saliva specific IgG antibodies in all study districts is consistent with the prevalence of this species (the main vector of CL in Mali) in all 3 districts [29]. Additionally, a similar level of antibodies in LST+ and LST- individuals may be a reflection of the low number of infected flies in sand fly populations, in Mali [7]. The difference the prevalence of LST positivity and the distribution of active cases of CL among the 3 districts may be due to a difference in the concentration of rodent reservoirs. Further studies are needed to fully understand the ecology and infection dynamics in both sand flies and reservoirs to elucidate the observed differences in the epidemiology of CL among the 3 districts.

In summary, the proportion of positive skin tests increased with age suggesting that the children are high risk to developing CL in Mali. Moreover, comparing 3 ecologically distinct areas in western, central and southeast Mali, we determined that the prevalence of LST positivity and active disease remains highest in the western part of Mali. Future steps will focus on the characterization of the *Leishmania* strains circulating in Diema area and correlating the specific human immune response to sand fly salivary proteins with CL outcome.

## Supporting Information

S1 Checklist STROBE Statement—Checklist of items included in reports of cross-sectional studies. (DOC)
