# Correction: Prevalence of Cutaneous Leishmaniasis in Districts of High and Low Endemicity in Mali

**DOI:** 10.1371/journal.pntd.0005379

**Published:** 2017-02-15

**Authors:** Bourama Traoré, Fabiano Oliveira, Ousmane Faye, Adama Dicko, Cheick A. Coulibaly, Ibrahim M. Sissoko, Samake Sibiry, Nafomon Sogoba, Moussa Brema Sangare, Yaya I. Coulibaly, Pierre Traore, Sekou F. Traore, Jennifer M. Anderson, Somita Keita, Jesus G. Valenzuela, Shaden Kamhawi, Seydou Doumbia

There is an error in the final sentence of the subsection “Assessing Anti-saliva antibodies levels”. The correct sentence is: Moreover, the median value of anti-salivary IgG levels were significantly higher (P = 0.0001) in LST+ compared to LST- study subjects (Fig 4B). Also, Fig 4 is missing the statistical significance value. The authors have provided a corrected version here.

**Fig 4:**
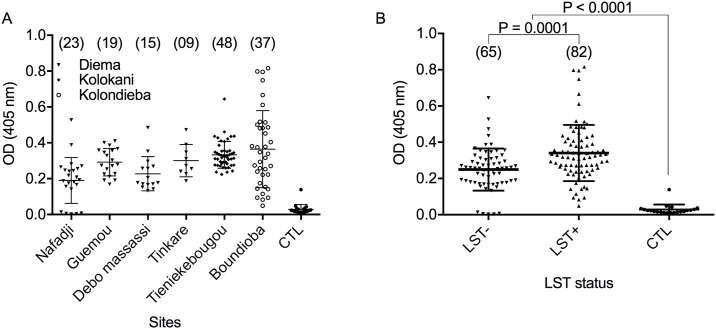
Optical density (OD) of antibodies against sand fly saliva measured by ELISA. A) Antibody levels in tested subjects from the 6 study villages. Sample size is shown in brackets. B) Overall antibody levels in leishmanin positive (LST+) and negative (LST-) study subjects. CTL, non-endemic controls from US healthy volunteers not exposed to *Leishmania*.
